# Effectiveness of a stepped-care intervention to prevent major depression in patients with type 2 diabetes mellitus and/or coronary heart disease and subthreshold depression: A pragmatic cluster randomized controlled trial

**DOI:** 10.1371/journal.pone.0181023

**Published:** 2017-08-01

**Authors:** Alide D. Pols, Susan E. van Dijk, Judith E. Bosmans, Trynke Hoekstra, Harm W. J. van Marwijk, Maurits W. van Tulder, Marcel C. Adriaanse

**Affiliations:** 1 Department of Health Sciences and EMGO Institute for Health and Care Research, VU University Amsterdam, Amsterdam, the Netherlands; 2 Department of General Practice & Elderly Care Medicine and EMGO Institute for Health and Care Research, VU University Medical Center, Amsterdam, the Netherlands; 3 Department of Epidemiology and Biostatistics, VU University Amsterdam, Amsterdam, the Netherlands; 4 CLAHRC Greater Manchester and NIHR School for Primary Care Research, the University of Manchester, Manchester, United Kingdom; Florida International University Herbert Wertheim College of Medicine, UNITED STATES

## Abstract

**Purpose:**

Given the public health significance of poorly treatable co-morbid major depressive disorders (MDD) among patients with type 2 diabetes mellitus (DM2) and coronary heart disease (CHD), we need to investigate whether strategies to prevent the development of major depression could reduce its burden of disease. We therefore evaluated the effectiveness of a stepped-care program for subthreshold depression in comparison with usual care in patients with DM2 and/or CHD.

**Methods:**

A cluster randomized controlled trial, with 27 primary care centers serving as clusters. A total of 236 DM2 and/or CHD patients with subthreshold depression (nine item Patient Health Questionnaire (PHQ-9) score ≥ 6, no current MDD according to DSM-IV criteria) were allocated to the intervention group (N = 96) or usual care group (n = 140). The stepped-care program was delivered by trained practice nurses during one year and consisted of four sequential treatment steps: watchful waiting, guided self-help, problem solving treatment and referral to the general practitioner. The primary outcome was the 12-month cumulative incidence of MDD as measured with the Mini International Neuropsychiatric Interview (MINI). Secondary outcomes included severity of depression (measured by PHQ-9) at 3, 6, 9 and 12 months.

**Results:**

Of 236 patients (mean age, 67,5 (SD 10) years; 54.7% men), 210 (89%) completed the MINI at 12 months. The cumulative incidence of MDD was 9 of 89 (10.1%) participants in the intervention group and 12 of 121 (9.9%) participants in the usual care group. We found no statistically significant overall effect of the intervention (OR = 1.21; 95% confidence interval (0.12 to 12.41)) and there were no statistically significant differences in the course or severity of depressive symptoms between the two groups.

**Conclusions:**

This study suggest that Step-Dep was not more effective in preventing MDD than usual care in a primary care population with DM2 and/or CHD and subthreshold depression.

## Introduction

Depression is projected to be the second cause of disease burden worldwide by 2030[1]. Depression and chronic illnesses such as diabetes mellitus type 2 (DM2) and coronary heart disease (CHD) often occur together and this can lead to a vicious circle, with each being a risk factor for the other[2]. Furthermore, such co-morbidity adversely affects self-care and medication adherence[3,4], quality of life[5], health status and increases mortality[6,7], and healthcare costs[8,9]. Subthreshold depression, i.e. clinically relevant depressive symptoms without fulfilling the criteria for major depressive disorder (MDD), is the strongest predictor for its onset[10,11]. In addition, people with both subthreshold depression and a history of depression are at even higher risk of another episode of MDD[12]. About a third of the patients with DM2 and/or CHD has subthreshold depression and more than 40% of those will develop MDD within two years[13–15].

Significant obstacles exist in the reduction of the burden of disease of depression. About one-third of those who receive treatment do not respond to current approaches, and over half of those who experience a first episode of MDD, will experience one or more recurrences[16]. Therefore, the burden of depression could be reduced considerably if the influx of new cases of depression could be prevented by early recognition and treatment of patients at risk, such as those with subthreshold depression. In comparison to control groups, preventative psychological interventions can overall reduce the incidence of MDD with a risk difference of 5%[10,17], but there is considerable heterogeneity.

Offering preventative psychological interventions in a stepped-care format is a possible solution, but the current evidence is both limited and conflicting[18,19]. Whereas some studies on prevention found beneficial effects of stepped-care as compared to usual care on the incidence of depression in the long-term[20–22], other studies found minor and short-term effects[23], or no beneficial effects at all[13,24,25]. Effects also seem to differ in various populations, and across efficacy and effectiveness studies (i.e., under practice circumstances)[26]. We investigated whether a pragmatic nurse-led stepped-care program is effective in reducing the incidence of MDD at 12-monts follow-up in comparison to usual care among patients with type 2 diabetes mellitus and/or coronary heart disease and subthreshold depression (Step-Dep trial).

## Methods

### Design

The Step-Dep study was a pragmatic cluster randomized controlled trial with a one-year follow-up. Step-Dep was conducted between January 2013 and November 2015, including recruitment and one year follow-up. The clusters consisted of primary care centers in the Netherlands, with 2000–8000 enlisted patients. Multiple general practitioners (GPs) at one location were considered one center and a single cluster.

### Trial registration and ethical approval

The study was performed in accordance with the declaration of Helsinki (2008) and the Dutch Medical Research involving Human Subjects Act (WMO). The protocol was approved by the medical ethics committee of the VU University Medical Centre (NL39261.029.12, registration number 2012/223), and registered in the Dutch Trial Register (NTR3715 http://www.trialregister.nl/trialreg/admin/rctview.asp?TC=3715) and published elsewhere[27].

### Setting

Primary care centers were recruited through local research networks of general practitioners. In total, 27 primary care centers with 53 general practitioners (GPs), 26 practice nurses (PN) and 128,280 enlisted patients consented to participate in the Step-Dep trial. Of these, 18 centers with 33 GPs, 18 PNs and 76,340 enlisted patients were located in urban areas, and 9 centers with 20 GPs, 8 PNs and 51,940 enlisted patients in rural areas. In order to resemble clinical daily practice as much as possible, only practice nurses already working at the participating centers administered the program.

### Randomization and blinding

Using a computer generated list of random numbers, a statistician blinded to the characteristics of the centers, performed the (cluster) randomization. Randomization was done at the level of the primary care center which corresponds to the participating practice nurse to avoid contamination between the treatment groups, and was stratified for size (less or more than 5000 patients). Due to the nature of the intervention, it was not possible to blind GPs or PNs to the intervention. All patients were informed about their treatment allocation before providing informed consent. All outcomes were assessed blinded to treatment allocation.

### Population

To identify eligible patients, the electronic patient record system at each primary care center was searched to select patients aged 18 years or more who had an International Classification of Primary Care (ICPC) diagnosis of DM2 and/or CHD (S1 Appendix). Participating GPs excluded patients with cognitive impairment, psychotic illnesses or a terminal illness, patients who were currently taking anti-depressant medication, had suffered the loss of a significant other in the past six months, or had a history of suicide attempt(s). Also, patients who were visually impaired, currently pregnant, had a bipolar disorder, or a borderline personality disorder or any difficulties completing written questionnaires or visiting the primary care center were excluded by the GP. All remaining patients received information about the study by mail, accompanied by an invitation from their own GP to participate and a PHQ-9 form to screen for depressive symptoms. Patients with a PHQ-9 score of six or higher were considered to have subthreshold depression[28,29]. After informed consent for a telephone interview, these patients were contacted within two weeks by trained research assistants, who administered the Mini International Neuropsychiatric Interview (MINI); a structured interview based on the criteria for MDD according to the Diagnostic and Statistical Manual of Mental Disorders, Fourth Edition (DSM-IV)[30,31]. All patients diagnosed with current MDD according to the MINI were excluded. Remaining patients were considered eligible to participate and received detailed information about the study together with an informed consent form by mail. Patients who returned a signed informed consent form were included in the study.

### Intervention

Step-Dep was modelled after the effective stepped-care intervention by van ‘t Veer-Tazelaar et al[20]. It consisted of four steps that increased in treatment intensity. All steps had a duration of three months; at the end of each step, depressive symptoms were evaluated by the PN using the PHQ-9[28,32]. Subsequently, when a patient had a PHQ-9 score of 6 or higher, more intensive steps were initiated. A PHQ-9 score below six, resulted in a period of watchful waiting. Patients showing recurrent subthreshold depressive symptoms (i.e. a PHQ-9 ≥ 6) after a period of remission (i.e. a PHQ-9 < 6) were offered the next sequential step they had not yet received. All steps of the intervention were implemented by the PN who coordinated the intervention and consulted the GP when necessary.

Step 1 consisted of watchful waiting starting with an introductory meeting with the PN. In this step, no active care was provided, because spontaneous recovery from subthreshold depressive symptoms occurs frequently[20].

Step 2 entailed a written guided self-help course that was especially developed to reduce depressive symptoms in patients with a chronic medical condition[33]. During this step, the PN called the patient every other week to monitor progress and motivate the patient.

Step 3 consisted of problem solving treatment (PST) provided by the PN. PST is a brief, proven effective cognitive behavioral intervention to treat depressive symptoms by focusing on practical skill building[34,35]. In Step-Dep, it consisted of a maximum of 7 sessions during 12 weeks. During the treatment, the stages of problem solving were explained and applied to problems a patient experienced in daily life.

In Step 4, patients were referred to their GP. This was initiated when subthreshold depression was still present after completing PST, or when the patient was diagnosed with MDD or expressed suicidal ideation according to the DSM-IV or DSM-V at any time during the intervention. If necessary, patients were referred to specialized mental health care or prescribed anti-depressant medication.

### Training of the practice nurses

Practice nurses in the intervention arm received a two-day training. This training focused on how to implement the stepped-care program, how to provide guidance with the self-help course using motivational interviewing techniques and how to provide the PST. The training was developed and provided by a qualified trainer in collaboration with team members (SVD and AP). During the trial, all practice nurses were regularly supervised by the training staff and they could contact the training staff to discuss any questions or problems. Additional information about the training has been previously provided[27].

### Usual care

Patients in the usual care condition had unrestricted access to care as normally provided according to existing Dutch clinical guidelines by their GP[36]. GPs and PNs working in usual care centers did not receive any additional training or detailed protocol information about Step-Dep. Because of medical ethical considerations, participation of patients was reported to their GP.

### Outcomes

The primary outcome was the cumulative incidence of MDD according to the DSM-IV, as measured with the MINI at 6 and 12 months of follow-up. Trained research assistants who were blinded to group allocation administered the MINI by telephone. The MINI is considered a reliable and valid instrument to diagnose MDD[30]. With an administration time of approximately 15 minute, the MINI has become the structured interview of choice for psychiatric evaluation in many clinical trials and epidemiological studies.

Secondary outcomes included depression severity and anxiety. Depression severity was measured by the PHQ-9 (range 0–27 with higher scores indicating more severe depression)[28]. The PHQ-9 is a widely used and validated instrument and performs well in patients with chronic medical illnesses[29,37]. Anxiety was measured by Hospital Anxiety and Depression Scale Anxiety (HADS-A; range 0–21 with higher scores indicating more severe anxiety)[38]. Depression severity and anxiety were measured at baseline and at 3, 6, 9 and 12 months using web-based questionnaires. When patients did not have access to the internet or preferred questionnaires on paper, these were provided.

Patient characteristics were assessed at baseline and included demographics (gender, age, marital status (together with spouse or alone), level of education (low, average, high), excessive alcohol use (more than 10 standard units per week for men and 5 units per week for women according to the Dutch standards[39]), current smoking behavior (yes/no), body-mass index (BMI) based on self-reported weight and height, exercise behavior (cut off for healthy exercise: 10 minutes a day, five days a week[40]) and ethnic origin (Dutch or non-Dutch). Additionally, we measured the number of depressive episodes in the past and the age of onset of the first depressive episode using a subset of the Diagnostic Interview Schedule (DIS)[41], the presence of co-morbid chronic illnesses using the self-reported Dutch Questionnaire Chronic Illnesses[42], locus of control (range 0–20, higher scores indicating a more external locus of control)[43,44], and social support (range 0–48, higher scores indicating more perceived social support)[45] at baseline.

### Sample size

Based on previous findings, we expected that, without any intervention, approximately 30% of patients would develop an MDD within one year follow-up, and that half of all new cases could be prevented by the Step-Dep intervention[10,20,46]. Thus, this trial was powered to detect a difference of 15% in the incidence of depression between both treatment groups. The power calculation was corrected for clustering within the multilevel setting at three levels (primary care centers, patients and repeated measurements). Assuming measurements are clustered within patients with an Intra Class Correlation Coefficient (ICC) of .45 and patients within the primary care centers with an ICC of .05, we needed a total of 177 patients, using 80% power and an alpha of 5%. After allowance for 25% attrition, a sample size of 236 patients (118 patients in each group) was needed.

### Statistical analyses

All analyses were conducted according to the intention to treat principle. First, all baseline variables were described; continuous variables as means (SD) and categorical variables as percentages. Secondly, the effectiveness of the intervention on the primary and secondary outcomes over time was analyzed with mixed models for longitudinal data; linear mixed models were used for continuous outcome variables and logistic mixed models for binary outcome variables. Mixed model analyses take the dependence of the repeated measurements into account, while maximizing the use of information that is present in the data without having to impute when data are missing at random (MAR)[47,48]. For each outcome an overall effect over time and separate effects at different time points were estimated by taking time into account as a categorical variable (with four categories: 0–3 months, 3–6 months, 6–9 months and 9–12 months of follow-up)[47,49].

The main analyses consisted of fully corrected models that were corrected for baseline values of the respective outcome and additionally included the covariates gender[2], age[50], and any other possible confounding variable on which the treatment groups differed at baseline (marital status, employment status, level of education, co-existence of DM2 and CHD, alcohol use, number of depressive episodes in history and age of onset of depression). The absolute baseline differences were judged by the researchers, rather than statistically tested, since relying on statistical testing of baseline differences ignores the prognostic strength of confounders[51].

## Results

### Participants

**Fig 1:**
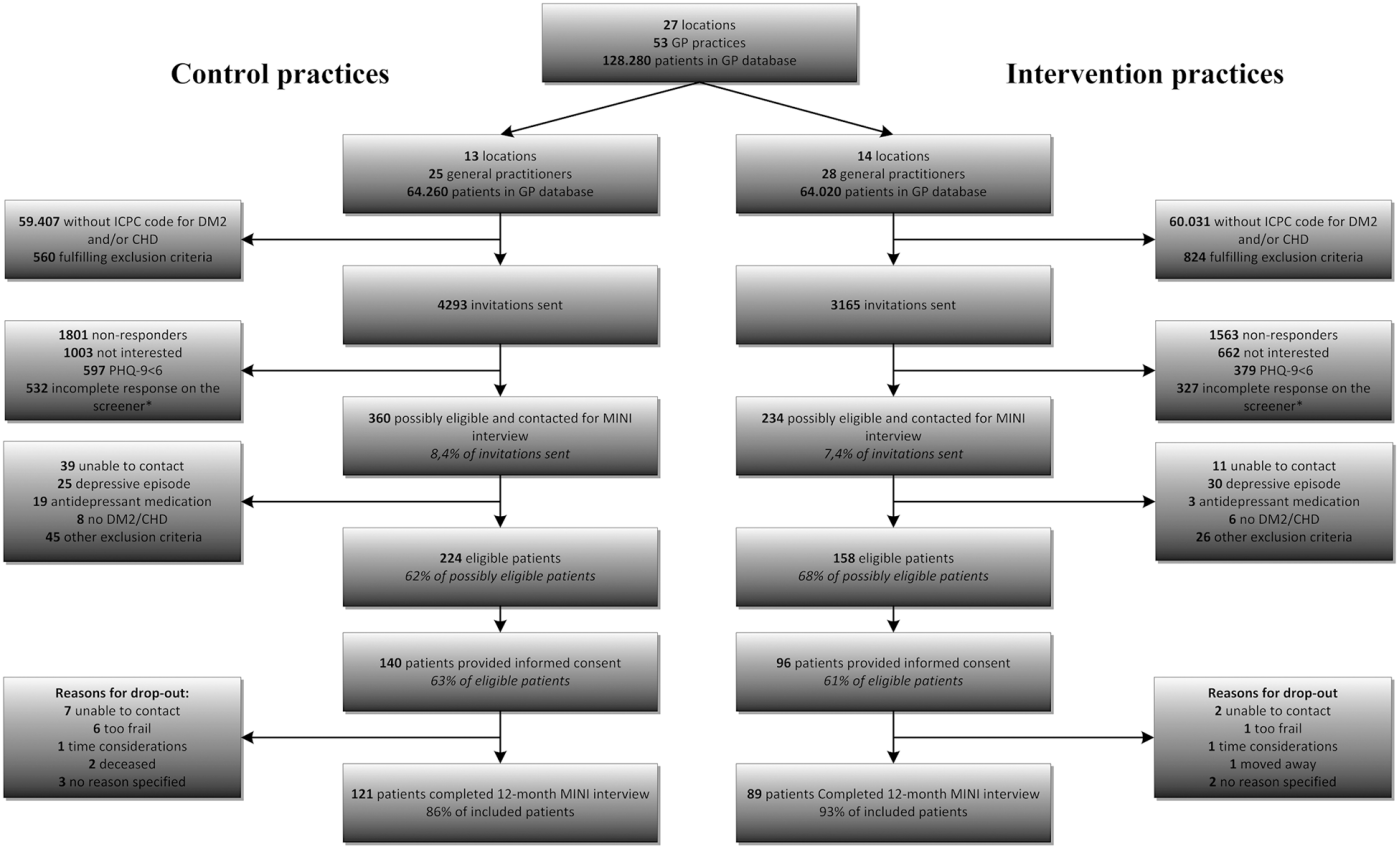
CONSORT participant flow diagram. Abbreviations: DM2, diabetes mellitus type 2; CHD, coronary heart disease; GP, general practitioner; ICPC, International classification of primary care; PHQ-9, Patient Health Questionnaire-9. *data on screener missing on either PHQ-9 scores, personal data or if interested.

“Fig 1” shows the sampling of the study participants. In total, 7458 patients were selected by their GPs as potentially eligible to participate in the Step-Dep study of whom 4094 (55%) returned the PHQ-9, and 594 (8%) had a score of 6 or more on the PHQ-9 and were interested in participating. Based on the MINI interviews, 382 patients (5%) were eligible to participate of whom 236 (3%) gave informed consent to participate in the study. Of these, 140 patients (63% of eligible patients from usual care centers) were included in the usual care group and 96 patients (61% of eligible patients from intervention centers) were included in the intervention group. Of all participants in both groups, 209 patients and 210 patients (89%) completed the MINI interview at six and 12 months respectively.

The mean age of the total sample was 67.5 years (standard deviation (SD) 10.0), 107 participants (45%) were female and the mean baseline PHQ-9 score in the total sample was 9.4 (SD 3.2). The baseline characteristics of all participants are summarized in “Table 1”.

**Table 1:** Patients’ baseline characteristics at baseline in intervention group, care as usual group and total sample.

Characteristics	Intervention (N = 96)	Care as usual (N = 140)	Total sample (N = 236)
Female	42 (43.8)	65 (46.4)	107 (45.3)
Age, mean (SD)	67.8 (9.2)	67.3 (10.5)	67.5 (10.0)
Marital status			
Married/living together	55 (57.3)	67 (47.9)	122 (51.7)
Single/divorced/widowed	35 (36.5)	63 (45)	98 (41.5)
Not reported	6 (6.3)	10 (10.4)	16 (6.8)
Both parents born in the Netherlands	74/90 (82.2)	112/130 (86.2)	186/220 (84.5)
Rural residential area	42 (43.8)	57 (40.7)	99 (41.9)
Unemployed/sick	12/90 (13.3)	14/130 (10.8)	26/220 (11.8)
Level of education			
Low	33 (34.4)	56 (40)	89 (37.7)
Average	22 (22.9)	38 (27.1)	60 (25.4)
High	35 (36.5)	36 (25.7)	71 (30.1)
Not reported	6 (6.3)	10 (7.1)	16 (6.8)
Diabetes Mellitus type 2 (DM2)	60 (62.5)	90 (64.3)	150 (63.6)
Coronary Heart Disease (CHD)	58 (60.4)	90 (64.3)	148 (62.7)
DM2 and CHD	22 (22.9)	40 (28.6)	62 (26.3)
Nr of chronic diseases, median (25^th^ -75^th^ percentile)	3 (2–5)	3 (2–5)	3 (2–5)
DM2 treated with insulin or oral medication	42/57 (73.7)	64/83 (77.1)	106/140 (75.7)
CHD treated with chronic medication	46/54 (85.2)	65/85 (76.5)	111/139 (79.9)
Current smoker	16/90 (17.8)	23/129 (17.8)	39/219 (17.8)
Alcohol use above norm	29/90 (32.2)	34 /129(26.4)	63/219 (28.8)
Exercise under norm	56/90 (62.2)	85/129 (65.9)	141/219 (64.4)
BMI, mean (SD)	29.4 (6.8)	28.5 (5.6)	28.9 (6.1)
Locus of Control, mean (SD)	8.3 (4.2)	7.6 (4.1)	7.9 (4.2)
Social support, mean (SD)	35.8 (9.0)	36.7 (9.5)	36.3 (9.2)
Dysthymia	6 (6.3)	7 (5.0)	13 (5.5)
Nr of depression in history			
0	35 (36.5)	65 (46.4)	100 (42.4)
1	14 (14.6)	11 (7.8)	25 (10.6)
2 or more	40 (41.7)	43 (30.7)	83 (35.2)
Not reported	7 (7.3)	21 (15)	28 (11.9)
Onset of depression after age of 55	38/89 (42.7)	63/121 (52.1)	101/210 (48.1)
PHQ-9 at baseline, mean (SD)	9.5 (3.1)	9.3 (3.2)	9.4 (3.2)
Depression HADS, mean (SD)	6.9 (3.9)	6.1 (3.7)	6.5 (3.8)
Anxiety HADS, mean (SD)	6.9 (3.7)	6.3 (3.9)	6.5 (3.8)

Figures are numbers (percentage) unless stated otherwise; Abbreviations: BMI = Body Mass Index; EQ-5D-5L = Euroqol 5 dimensions 5 levels, PHQ-9, Patient Health Questionnaire-9; HADS, Hospital Anxiety and Depression Scale; SD, Standard Deviation.

### Uptake of the intervention

Of the 96 patients who were included in the intervention group, 90 patients (94%) started the intervention. In total, 60 patients (63%) received only watchful waiting (step 1), and 25 (26%) patients received guided self-help (step 2). Another 11 patients were offered the guided self-help course, but declined. Nine patients (9%) started PST (step 3), and 6 (6%) declined. Three patients were referred to the GP at the end of the program, and 5 other patients were referred to the GP during another treatment step. In total, 25 patients (26%) dropped out from the intervention due to frailty (n = 7), time restraints (n = 2), lack of motivation (n = 7), moving away (n = 2), or for unknown reasons (n = 7).

### Effectiveness of intervention

The number of participants with a MDD at 6 months was 5 of 84 (6.0%) in the intervention group and 10 of 125 (8.0%) in the usual care group. The cumulative incidence of MDD at 12 months was 9 of 89 (10.1%) participants in the intervention group and 12 of 121 (9.9%) participants in the usual care group (“Table 2”). There was no statistically significant overall treatment effect over 12 months of the intervention (OR = 1.21; 95% confidence interval (0.12 to 12.41)). Due to the low incidence of MDD, the analyses of the differences between the different time points did not converge. Therefore, only overall results are presented.

**Table 2:** Results of the mixed model analyses.

**Cumulative incidence of depression (n/N) %**	**Intervention**	**Care as usual**	Corrected analyses*		**Crude analyses**	
**Baseline**	0	0	**OR (95%CI)**	**P-value**	**OR (95%CI)**	**P-value**
**T6**	(5/84) 6.0	(10/125) 8.0	Did not converge	n.e	Did not converge	n.e
**T12**	(9/89) 10.1	(12/121) 9.9	Did not converge	n.e	Did not converge	n.e
**Overall effect**	n.a	n.a	1.21 (0.12; 12.41)	0.87	1.05 (0.05; 22.47)	0.98
**PHQ mean (SD)**	**Intervention**	**Care as usual**	Corrected analyses*		**Crude analyses**	
**Baseline**	9.53 (3.14)	9.28 (3.23)	**B (95%CI)**	**P-value**	**B (95%CI)**	**P-value**
**T3**	6.68 (4.55)	6.58 (4.21)	-0.42 (-1.54; 0.71)	0.47	0.20 (-1.05; 1.46)	0.75
**T6**	6.10 (4.43)	6.12 (4.41)	-0.38 (-1.50; 0.75)	0.51	0.06 (-1.19; 1.30)	0.93
**T9**	6.28 (4.31)	6.46 (4.51)	-0.52 (-1.65; 0.61)	0.37	-0.20 (-1.45; 1.05)	0.75
**T12**	6.60 (5.23)	6.29 (4.46)	-0.12 (-1.22; 0.99)	0.84	0.22 (-1.00; 1.44)	0.72
**Overall effect**	n.a	n.a	-0.02 (-0.93; 0.89)	0.97	0.01 (-0.90; 0.91)	0.99
**Perceived recovery (%)**	**Intervention**	**Care as usual**	Corrected analyses*		**Crude analyses**	
**Baseline**	n.a	n.a	**OR (95%CI)**	**P-value**	**OR (95%CI)**	**P-value**
**T3**	40.3%	49.5%	0.72 (0.26; 1.96)	0.52	0.49 (0.19; 1.31)	0.16
**T6**	48.8%	45.5%	2.05 (0.75; 5.60)	0.16	1.35 (0.52; 3.54)	0.54
**T9**	55.0%	48.7%	2.09 (0.76; 5.69)	0.15	1.69 (0.65; 4.43)	0.28
**T12**	55.6%	58.1%	1.16 (0.42; 3.20)	0.77	0.90 (0.34; 2.37)	0.83
**Overall effect**	n.a	n.a	1.37 (0.67; 2.80)	0.39	1.01 (0.51; 2.00)	0.97
**HADS-A mean (SD)**	**Intervention**	**Care as usual**	Corrected analyses*		**Crude analyses**	
**Baseline**	6.91 (3.74)	6.25 (3.90)	**B (95%CI)**	**P-value**	**B (95%CI)**	**P-value**
**T3**	6.35 (4.04)	6.29 (3.97)	-0.32 (-1.18; 0.53)	0.46	0.11 (-1.04; 1.26)	0.85
**T6**	5.70 (4.10)	6.63 (4.00)	-1.05 (-1.90; -0.20)	0.02	0.77 (-1.91; 0.38)	0.19
**T9**	6.16 (4.24)	6.03 (4.04)	-0.52 (-1.37; 0.33)	0.23	-0.07 (-1.22; 1.07)	0.90
**T12**	5.77 (4.69)	5.83 (3.99)	-0.52 (-1.38; 0.34)	0.23	-0.27 (-1.43; 0.88)	0.64
**Overall effect**	n.a	n.a	-0.31 (-1.29; 0.65)	0.53	0.01 (-0.97; 0.99)	0.99
**HADS-D mean (SD)**	**Intervention**	**Care as usual**	Corrected analyses*		**Crude analyses**	
**Baseline**	6.93 (3.87)	6.11 (3.73)	**B (95%CI)**	**P-value**	**B (95%CI)**	**P-value**
**T3**	6.14 (4.16)	6.21 (3.87)	-0.30 (-1.13; 0.52)	0.47	0.09; (-1.03; 1.21)	0.87
**T6**	5.82 (3.79)	5.75 (4.03)	-0.22 (-1.04; 0.60)	0.60	0.19 (-0.93; 1.30)	0.74
**T9**	6.36 (4.04)	6.07 (4.08)	-0.24 (-1.07; 0.58)	0.56	0.17 (-0.95; 1.28)	0.77
**T12**	6.09 (4.20)	6.11 (4.22)	-0.42 (-1.25; 0.41)	0.32	-0.07 (-1.19; 1.05)	0.90
**Overall effect**	n.a	n.a	0.18 (-0.79; 1.16)	0.71	0.27 (-0.68; 1.22)	0.58

Abbreviations: 95%CI, 95% Confidence Interval; HADS-A, Hospital Anxiety and Depression Scale-Anxiety; HADS-D, Hospital Anxiety and Depression Scale-Depression; n.a, not applicable; n.e, not estimated; PHQ-9, Patient Health Questionnaire-9;

*Corrected for: baseline values of the outcome, age, gender, marital status, employment status, level of education, co-existence of DM2 and CHD, alcohol use, number of depressive episodes in history and age of onset of depression. The baseline value of the outcome is not added as an extra variable in the corrected analyses of the overall effects since it is already incorporated in the crude overall analyses.

**Fig 2:**
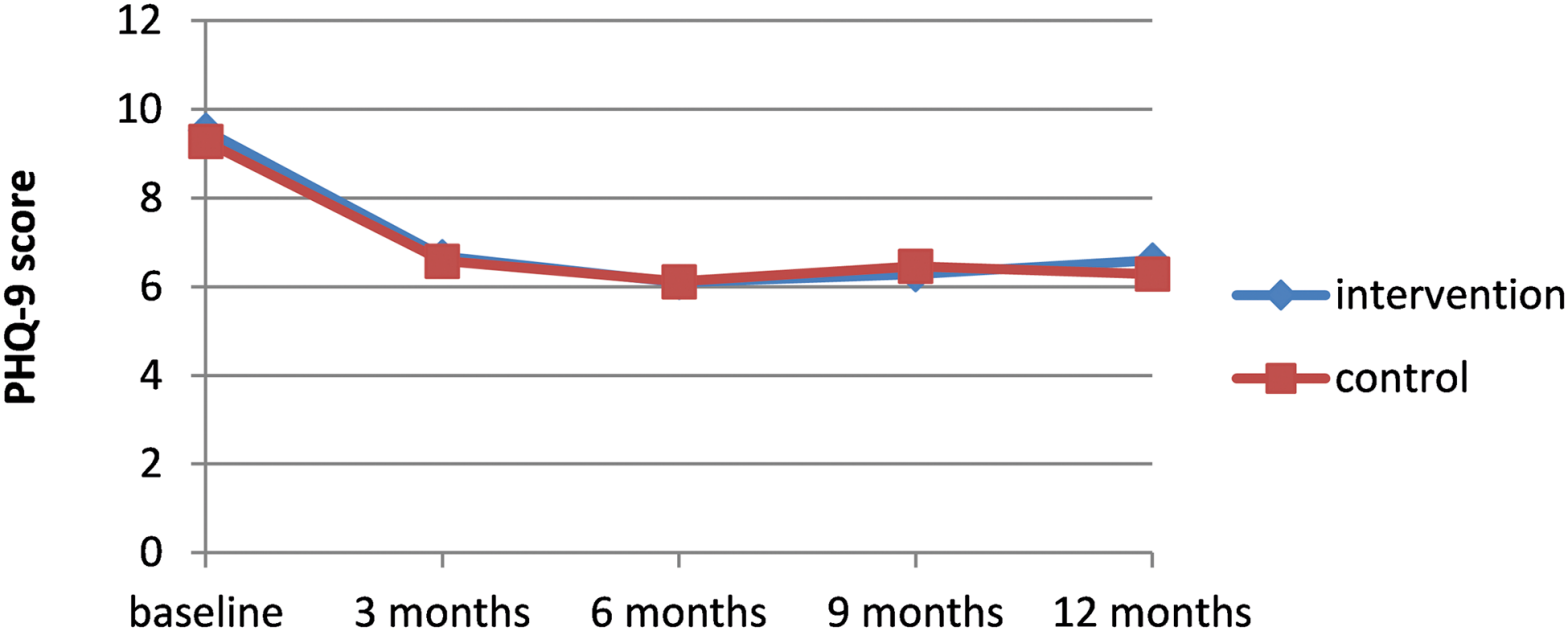
12-month course of depression severity as measured with the PHQ-9.

In both groups, the PHQ-9 score decreased almost 3 points between baseline and 3 months. After 3 months, PHQ-9 scores remained quite stable in both groups (“Fig 2”). There were no significant differences in PHQ-9 scores between the study groups at any time point. The course of PHQ-9 scores over time did not differ significantly between the groups. The anxiety scores in the intervention group at 6 months of follow-up were statistically significantly lower than in the usual care group. However, there were no statistically significant differences at the other time points nor a statistically significant difference in the course of anxiety symptoms over time between the groups.

## Discussion

### Main findings

This study found no statistically significant difference over 12 months between the Step-Dep intervention and usual care in the onset of MDD in primary care patients with DM2 and/ or CHD who screened positively for subthreshold depression. It showed that there were no statistically significant differences in secondary outcomes (PHQ-9, HADS-D and perceived recovery) between groups, and the symptoms in both groups showed virtually the same course. However, there was a statistically significant difference in anxiety scores at 6 months of follow-up, but this difference was not clinically relevant. Also, this was not seen at any other time point nor was the overall effect in anxiety scores statistically significant. Therefore, we think that this was most probably due to multiple testing.

### Findings in relation to other studies

Step-Dep is the first study that evaluates the effectiveness of a stepped-care program to prevent MDD in comparison with usual care in patients with DM2 and/or CHD and subthreshold depression in a primary care setting. Our negative findings are in line with preventative stepped-care studies among primary care patients with subthreshold depression and/or anxiety in Hong Kong[25], in community dwelling older adults[23], and older adults in general practice[24], but in contrast with studies in other groups of older adults[20,21], and in visually impaired older adults[22].

The first potential explanation for the heterogeneity in findings is that the incidence of depression in the current studies was lower than in previous studies[20–22]. This may indicate that the risk of developing a MDD in our study was lower than in those study populations. More stringent inclusion criteria might have prevented this. Van ‘t Veer et al[20], for example, only included respondents who had elevated depression scores on two consecutive occasions. Moreover, the cut-off score of 6 on the PHQ-9 that we used for this study may have been too low; a score of eight or higher might have been more appropriate[52]. A higher cut-off may be necessary, because symptoms of depression and DM2 and/ or CHD partly overlap (e.g. fatigue, change of appetite), which potentially results in a high risk of over-diagnosing (subthreshold) depression in this group[53]. Also, stepped-care may be more effective patients with more severe symptoms[54]. However, we do believe to have included an adequate patient population as patients had an average PHQ-9 score of 9.4 at inclusion and about 58% of the total sample had a (self-reported) history of depression. Secondly, in our study, fewer patients than expected were eligible for the more intensive treatment steps due to their low PHQ-9 scores of 6.7 on average at three months after baseline measurements, whereas the cut-off for a more intensive treatment step was set at 6 or higher. The drop in PHQ-9 scores between baseline and three months follow-up in both groups exceeds the expectations of spontaneous recovery alone[20]. It is not likely to be caused by treatment either. In the intervention condition, patients were offered watchful waiting during this period. In the usual care condition, it is unlikely that notifying general practitioners which participants met criteria for subthreshold depression led to any treatment in this period, because screening for depression alone does not lead to changes in the management of depression[55]. Additionally, the Dutch clinical Guidelines advice an initial period of watchful waiting for subthreshold depression[36]. Perhaps the decrease in depressive symptoms is partly caused by attention or patients’ self-insight into their mental problems. Thirdly, a considerable proportion of patients (29%) did not want to start one or more of the treatment steps. The treatment delivery rates were similar to those in other trials that did not find a significant effect[23–25], but considerably lower than in trials that did find a significant effect on depression outcomes[20–22]. Thus, the uptake of the intervention may have influenced the outcomes. This may indicate that our program did not match the need for care in this population. Finally, depressive and anxiety symptoms slightly improve over time in both groups. This might indicate that usual care is already of reasonable quality and, therefore, the room for improvement for new interventions over usual care is limited.

### Strengths and limitations

The most important strengths of Step-Dep were its randomized controlled trial design, the use of reliable and clinically meaningful outcome measures, and the low dropout rate during one-year follow-up. Another strength is its pragmatic approach; the intervention was tested in a real life setting, which increases the generalizability of the findings of the study.

This study also has some limitations. First, the combination of an unforeseen low incidence of MDD and a relatively small size made this study underpowered to rule out a clinically relevant difference between groups. However, given the lack of statistically significant or clinically relevant differences in all secondary outcome measures, it seems unlikely that the intervention was superior to usual care in preventing MDD. Second, neither patients nor healthcare providers could be blinded to the intervention. Third, from all DM2 and/ or CHD patients who were initially invited to be screened, only 21% returned a completed PHQ-9 screening form and were interested in participation. Fourth, due to the cluster randomization and ethical considerations, we had to inform patients before inclusion which treatment group they would be in if they participated. This could have resulted in selection bias. However, the percentages of invited and eligible patients are comparable between groups. Also, there were no clinically relevant differences in key baseline characteristics between the two treatment groups, making it unlikely that the groups originated from different patient populations. Finally, some adjustments were made to the published protocol[27]. The implemented inclusion procedure did not contain a preliminary screening with the two-item Patient Health Questionnaire (PHQ-2), since it resulted in a large number of false positive screening results, making it not feasible to interview all patients with a positive screening result by telephone. Biomedical outcome measures (blood pressure, low-density lipoprotein cholesterol and glycosylated haemoglobin) were not measured at baseline and 12 months of follow-up, since patients had to pay for these measurements outside their regular check-ups themselves. The HADS-D and the perceived recovery scale were added before the inclusion of patients started which was approved in an amendment by the medical ethical review board.

## Conclusions

In conclusion, this study suggest that Step-Dep was not superior to care as usual in the prevention of MDD in a population with DM2 and/or CHD that screened positively for subthreshold depression. Widespread implementation of Step-Dep in clinical practice in patients screened for subthreshold depression is, therefore, currently not recommended. We recommend further research to evaluate the effectiveness of targeting interventions to patients with more severe depressive symptoms on two consecutive occasions, but only after further exploring their need for care. Our results feed the ongoing debate on the feasibility of stepped-care and screening on (subthreshold) depression in the chronically ill.

## Supporting information

S1 Appendix International classifications of primary care codes. (DOCX)

S2 Appendix CONSORT-checklist. (DOC)

S3 Appendix Original protocol METC October 2013. (DOCX)
