# Supplementary material for: Effectiveness of a stepped-care intervention to prevent major depression in patients with type 2 diabetes mellitus and/or coronary heart disease and subthreshold depression: A pragmatic cluster randomized controlled trial
Source: PLoS One. 2017 Aug 1;12(8):e0181023. doi: 10.1371/journal.pone.0181023 (PMC5538642; doi:10.1371/journal.pone.0181023)
Supplement: S1 Appendix — (DOCX) [file pone.0181023.s001.docx]

**S1 Appendix 1: International Classifications of Primary Care codes**

| **ICPC code** | **Description** |
| --- | --- |
| T90 | Diabetes |
| T90.02 | Diabetes Mellitus type 2 |
| K74 | Angina pectoris |
| K74.01 | Instable angina pectoris |
| K74.02 | Stable angina pectoris |
| K75 | Acute myocardial infarction |
| K76 | Other/chronic ischemic disease |
| K76.01 | Coronary sclerosis |
| K76.02 | Previously experienced myocardial infarction (> 4 weeks ago) |
