# Supplementary material for: Effectiveness of a stepped-care intervention to prevent major depression in patients with type 2 diabetes mellitus and/or coronary heart disease and subthreshold depression: A pragmatic cluster randomized controlled trial
Source: PLoS One. 2017 Aug 1;12(8):e0181023. doi: 10.1371/journal.pone.0181023 (PMC5538642; doi:10.1371/journal.pone.0181023)
Supplement: S3 Appendix — (DOCX) [file pone.0181023.s003.docx]

**Cost effectiveness of a stepped-care program to prevent depression among primary care patients with type 2 diabetes and/or coronary heard disease and sub threshold depression**

**(Step-Dep)**

**version 4.0 (Oktober 2013)**

**PROTOCOL TITLE** ‘Cost effectiveness of a stepped-care program for depression among primary care patients with type 2 diabetes and/or coronary heard disease and sub threshold depression’

| **Protocol ID** | **NL392610291**  **METC ref: 12/223** |
| --- | --- |
| **Short title** | **Step-Dep** |
| **Version** | **3** |
| **Date** | **20-02-2013** |
| **Coordinating investigator/project leader** | ***Maurits van Tulder***  ***De Boelelaan 1085 HV***  ***Amsterdam***  ***020 598 6587***  [***maurits.van.tulder@vu.nl***](mailto:maurits.van.tulder@vu.nl) |
| **Principal investigator (in Dutch: hoofdonderzoeker/uitvoerder)** | ***Susan van Dijk***  ***De Boelelaan 1085 HV***  ***Amsterdam***  ***020 5986865***  [***s.e.m.van.dijk@vu.nl***](mailto:s.e.m.van.dijk@vu.nl)  ***Lidy Pols***  ***De Boelelaan 1085 HV***  ***Amsterdam***  ***06 28928497***  ***a.d.pols@vu.nl*** |
|  |  |
| **Sponsor (in Dutch: verrichte/opdrachtgever)** | ***Department of Health Sciences and EMGO^+^ institute***  **VU University Amsterdam**  **Van der Boechorststraat 7**  **1081 BT Amsterdam**  **020 444 8180** |
|  |  |
| **Independent physician(s)** | ***Karolien Biesheuvel-Leliefeld,***  ***van der Boechorststraat 7***  ***1081 BT Amsterdam***  **020-5986865,** [***k.biesheuvel@vumc.nl***](mailto:k.biesheuvel@vumc.nl) |

**PROTOCOL SIGNATURE SHEET**

| **Name** | **Signature** | **Date** |
| --- | --- | --- |
| **For non-commercial research,**  **Head of Department:**  ***Prof. dr. J.C. Seidell,***  ***Professor Nutrition and Health***  ***Afdelingshoofd gezondheidswetenschappen*** |  |  |
| **Coordinating Investigator/Project leader/Principal Investigator:**  ***S.E.M. van Dijk, MSc,***  ***PhD Candidate Health Economics and Health Technology Assessment.***  ***A.D. Pols***  ***PhD Candidate General Medicine and General Practicioner trainee*** |  |  |

**TABLE OF CONTENTS**

1. INTRODUCTION AND RATIONALE 8

2. OBJECTIVES 10

3. STUDY DESIGN 11

4. STUDY POPULATION 12

4.1 Population (base) 12

4.2 Inclusion criteria 12

4.3 Exclusion criteria 12

4.4 Sample size calculation 13

5. TREATMENT OF SUBJECTS 14

5.1 Investigational product/treatment 14

5.2 Use of co-intervention (if applicable) 15

5.3 Escape medication (if applicable) 15

6. INVESTIGATIONAL MEDICINAL PRODUCT 16

7. METHODS 17

7.1 Study parameters/endpoints 17

7.1.1 Main study parameter/endpoint 17

7.1.2 Secondary study parameters/endpoints (if applicable) 17

7.1.3 Other study parameters (if applicable) 17

7.2 Randomisation, blinding and treatment allocation 17

7.3 Study procedures 18

7.4 Withdrawal of individual subjects 20

7.4.1 Specific criteria for withdrawal (if applicable) 20

7.5 Replacement of individual subjects after withdrawal 20

7.6 Follow-up of subjects withdrawn from treatment 20

7.7 Premature termination of the study 20

8. SAFETY REPORTING 21

8.1 Section 10 WMO event 21

8.2 Adverse and serious adverse events 21

8.2.1 Suspected unexpected serious adverse reactions (SUSAR) 22

8.2.2 Annual safety report 22

8.3 Follow-up of adverse events 22

8.4 Data Safety Monitoring Board (DSMB) 22

9. STATISTICAL ANALYSIS 23

9.1 Descriptive statistics 23

9.2 Univariate analysis 23

9.3 Multivariate analysis 23

9.4 Interim analysis (if applicable) 24

10. ETHICAL CONSIDERATIONS 25

10.1 Regulation statement 25

10.2 Recruitment and consent 25

10.3 Objection by minors or incapacitated subjects (if applicable) 26

10.4 Benefits and risks assessment, group relatedness 26

10.5 Compensation for injury 26

10.6 Incentives (if applicable) 26

11. ADMINISTRATIVE ASPECTS AND PUBLICATION 27

11.1 Handling and storage of data and documents 27

11.2 Amendments 27

11.3 Annual progress report 27

11.4 End of study report 28

11.5 Public disclosure and publication policy 28

12. REFERENCES 29

13. APPENDIX 32

**LIST OF ABBREVIATIONS AND RELEVANT DEFINITIONS**

| **ABR** | **ABR form, General Assessment and Registration form, is the application form that is required for submission to the accredited Ethics Committee (In Dutch, ABR = Algemene Beoordeling en Registratie)** |
| --- | --- |
| **AE** | **Adverse Event** |
| **AR** | **Adverse Reaction** |
| **CA** | **Competent Authority** |
| **CCMO** | **Central Committee on Research Involving Human Subjects; in Dutch: Centrale Commissie Mensgebonden Onderzoek** |
| **CV** | **Curriculum Vitae** |
| **DSMB** | **Data Safety Monitoring Board** |
| **EU** | **European Union** |
| **EudraCT** | **European drug regulatory affairs Clinical Trials** |
| **GCP** | **Good Clinical Practice** |
| **IB** | **Investigator’s Brochure** |
| **IC** | **Informed Consent** |
| **IMP** | **Investigational Medicinal Product** |
| **IMPD** | **Investigational Medicinal Product Dossier** |
| **METC** | **Medical research ethics committee (MREC); in Dutch: medisch ethische toetsing commissie (METC)** |
| **(S)AE** | **(Serious) Adverse Event** |
| **SPC** | **Summary of Product Characteristics (in Dutch: officiële productinfomatie IB1-tekst)** |
| **Sponsor** | **The sponsor is the party that commissions the organisation or performance of the research, for example a pharmaceutical**  **company, academic hospital, scientific organisation or investigator. A party that provides funding for a study but does not commission it is not regarded as the sponsor, but referred to as a subsidising party.** |
| **SUSAR** | **Suspected Unexpected Serious Adverse Reaction** |
| **Wbp** | **Personal Data Protection Act (in Dutch: Wet Bescherming Persoonsgevens)** |
| **WMO** | **Medical Research Involving Human Subjects Act (in Dutch: Wet Medisch-wetenschappelijk Onderzoek met Mensen** |

**SUMMARY**

**Rationale:** Co-morbid depressive symptoms constitute a significant health problem among patients with type 2 diabetes mellitus (DM2) and/or coronary heart disease (CHD). Depressive symptoms increase the risk for major depression and are associated with poor compliance to treatment, adverse health outcomes and lower overall quality of life. Prevention of the development of major depression in high risk patients is expected to reduce the burden of depression for individuals and society.

**Objective**: This study aims to evaluate the cost-effectiveness of a nurse-led indicated stepped care program to prevent depression in comparison with usual care among DM2 and/or CHD primary care patients with subthreshold depressive symptoms.

**Study design:** An economic evaluation will be conducted from a societal perspective alongside a cluster-randomized controlled trial in approximately thirty general practices in the Netherlands. Randomisation takes place at the level of participating general practices. We aim to include 236 (118/118) participants who will either receive a nurse-led indicated stepped care program for depression or care as usual. There is a one year follow up, with measurements at baseline and after 3, 6, 9 and 12 months. An additional follow up will be performed at 24 and 36 months.

**Study population:** We aim to include adult patients (18 years or older) with DM2 and/or CHD who are treated in primary care and have subthreshold depression according to the Patient Health Questionnaire (PHQ-9).

**Intervention (if applicable)**: The stepped care program consists of four sequential but flexible treatment steps: watchful waiting, guided self-help treatment, problem solving treatment, and referral to the general practitioner for more intensive treatment.

**Main study parameters/endpoints:** The primary clinical outcome measure is the cumulative incidence of major depressive disorder after 12 months as measured with the Mini International Neuropsychiatric Interview (MINI). Other clinical outcomes include severity of depressive symptoms, quality of life, blood pressure, cholesterol and HbA1c. Costs will be measured from a societal perspective and include health care and lost productivity costs.

**Nature and extent of the burden and risks associated with participation, benefit and group relatedness:** Participating in this study will mean that patients have to fill in web-based questionnaires at baseline and at 3, 6, 9 and 12 months. Patients in the experimental condition will be offered a maximum of four sequential evidence based treatment steps. Patients in the control condition will be offered care as usual. No treatment will be denied to any participants nor will it be postponed. There is no additional medical risk present for any participants in this study. The experimental group will potentially have better treatment options for their depressive symptoms. Patients will be asked to participate in additional follow up at 24 and 36 months.

1. **INTRODUCTION AND RATIONALE**

Subthreshold depression, the presence of symptoms of depression without fulfilling the criteria for major depression, is the strongest predictor for the onset of major depression^1 2^. Currently, major depression is a substantial health problem trough out the industrialised world: It is ranked fourth worldwide in disease burden, and is expected in high-income countries to rank first in disease burden by the year 2030 ^3^. Among patients with type 2 diabetes (DM2) and/or coronary heart disease (CHD), also having a major depression is a particularly significant health problem, with an estimated 12 month prevalence of up to 20% ^4-6^ against approximately 6% in the Dutch community ^7^ and 3 to 9% in other high-income countrys ^8^ . Even more patients who suffer from DM2 also experience subthreshold depression, i.e. 25-40% ^9^. More than 40% of these patients develop major depression within two years ^9^. For patients with CHD and subthreshold depression similar estimates are found^10^.

Co-morbid depression has been shown to adversely affect self-care and medication adherence related to DM2 and CHD ^11 12^, to negatively impact quality of life ^13 14^ and to be associated with poor health outcomes and an increased risk of mortality ^15 16^. As a result of this, DM2 and CHD patients who also suffer from depression are using healthcare services more often than their non-depressed counterparts, which is associated to a substantial increase in health care related costs ^17-19^. In addition to this, having depression causes a large loss of productivity, which raises the total costs of depression even more^20^.

Unfortunately, it is extremely difficult to reduce these adverse effects of major depression once the disorder is present^21^. Therefore, prevention of the onset of major depression among high risk patients with diabetes and/or CHD and subthreshold depression may be promising to reduce the burden for both patients and society substantially.

Multiple meta analyses show that prevention of depression can indeed be successful. Using preventive interventions, a reduction of about 25 % in the incidence of major depression can be obtained^2 22^. Especially promising are the preventive interventions that are offered in Stepped- Care format. The aim of stepped-care interventions is to maximize the effectiveness of an intervention while making best use of available resources by tailoring the treatment to the patient’s preferences and by offering the least intensive treatment necessary. Using such a format is shown to reduce the incidence of depression by as much as 50%^22^. Moreover, recently an individual stepped-care program for depression was demonstrated to be effective in reducing the risk of the onset of depression among elderly individuals in the Netherlands.^23^

There is also evidence that stepped-care interventions can treat depression among patients with DM2 and CHD in primary care. Katon et. al. showed that stepped care for patients with chronic diseases was effective to treat depression ^24^ and later also to improve disease control ^25^. However, it remains unknown whether a stepped care program is cost-effective in comparison with usual care to prevent major depression in patients with a chronic illness and subthreshold depressive symptoms.

We hypothesize that the stepped care program reduces the incidence of depression and healthcare costs among primary care patients with diabetes and/or CHD and sub threshold depression.

1. **OBJECTIVES**

This study aims to evaluate the cost-effectiveness of a nurse-led indicated stepped-care program to prevent major depression among primary care patients with type 2 diabetes mellitus and/or coronary heart disease and subthreshold depression in comparison with usual care.

Primary research questions:

1. What are the effects of an integrated stepped-care care program in comparison with usual care after 1 year on the cumulative incidence of depression, severity of depression symptoms, and quality of life?

2. Is the stepped care program cost-effective in comparison with usual care from a societal perspective?

Other research questions that will be assessed are:

3. What are the effects of the stepped-care care program in comparison with usual care after 1 year on blood pressure, cholesterol and blood glucose (HbA1C)?

4. What is the uptake of the stepped care program? How satisfied are patients and care managers with the stepped care program? What are barriers and facilitators for implementation of a stepped care program?

5. What are the effects of the stepped-care program in comparison with usual care after 2 and 3 years on the cumulative incidence of depression, severity of depression symptoms, quality of life and on blood pressure, cholesterol and blood glucose (HbA1C)?6. Is the stepped care program cost-effective in comparison with usual care from a societal perspective after 2 and 3 years?

# STUDY DESIGN

An economic evaluation alongside a cluster randomised controlled trial with 1 year follow-up will be performed. After 24 and 36 months, participants will be approached again to participate in a long time follow-up. The cost-effectiveness of the stepped care program will be evaluated in comparison with usual care. Practices will be randomised to avoid contamination between the treatment groups. Practice nurses and GPs in intervention practices will be trained before the start of the study by qualified trainers.

Flow chart:

# STUDY POPULATION

## Population (base)

Patients will be eligible for this study if they are 18 years or older, have DM2 or CHD and have subthreshold depressive symptoms without fulfilling the criteria for major depression. To recruit patients we will make use of the academic network of GPs of the Department of General practice of the VUmc and other networks of general practitioners. Based on a pilot search in the Vumc academic network, we found that there are 11.000 patients of 18 years and older with diabetes and/or cardiovascular disease in the participating GP practices. We assume a response rate to the screening questionnaire of 40%. Based on our current experience, we expect that 15% of the responders will score 6 points or more on the PHQ-9(n=660). After excluding 10% of the remaining patients because they have a major depression according to the MINI (n=66), approximately 594 patients with subthreshold depression are potentially eligible for the study in the VUmc academic network. Based on these figures we are confident that we are able to include the number of patients needed according to our sample size calculation (n=236). However, to increase the feasibility of including enough patients, other general practitioner networks will also be requested to participate in the study.

## Inclusion criteria

- Diabetes type 2 and/or coronary heart disease (ICPC code T90 and/or ICPC code K74, K75 or K76);
- age 18 or older;
- treated for diabetes and/or coronary heart disease in primary care;
- subthreshold depression (PHQ-9 score ≥ 6 and MINI interview negative for major depressive disorder).

## Exclusion criteria

- major depressive disorderaccording to the MINI;
- Bipolar disorder
- cognitive impairment or dementia;
- psychotic illness;
- terminal illness;
- currently taking antidepressant medication;
- a history of suicide attempt(s);
- insufficient Dutch language skills;
- visual impairments or illiteracy;
- loss of significant other < previous 6 months; or
- pregnancy.

## Sample size calculation^[[1]](#footnote-1)^

This trial is powered to detect a difference of 15% in the cumulative incidence rates of MINI/DSM-IV depressive disorder between the conditions after 1 year. The incidence rate is expected to be 30% in the usual care group and 15% in the intervention group based on findings from earlier studies^2 22 23^. Using a standard sample size calculation while neglecting the fact that we have four repeated measurements clustered within patients and that these patients are clustered within general practitioners, 121 patients per group are needed, assuming a power of 0.8 and an alpha of 0.05. However, we need to correct for the fact that there is a multilevel setting with three levels: GP’s, Patients and measurements. Therefore we have first adjusted the 121 patients per group because we do not have one, but we have four measurements per patients. Assuming these are clustered with an intraclass correlation (ICC) of 0.45, seventy-one patients per group are needed. Next, we adjusted this figure further to the fact that patients are clustered within GP’s taking into consideration that we expect to include 30 GP’s in this trial. Assuming an ICC of 0.05 for clustering of patients within the 30 GP practices, we need a total of 177 patients. Finally, we adjusted for a dropout rate of 25%, which means that 236 patients (118 patients per group) need to be included in this trial. We used the following formula for the multi level sample size calculations:

M*n = N*[1+(n-1)*ICC] (1)

With M = number of clusters, n = number of patients per cluster; N = number of patients needed from the standard sample size calculation; ICC = intra-class correlation coefficient (measure for the amount of clustering).

From this formula, we can derive a formula for the efficiency factor in longitudinal designs given by:

EF: n/ [1+(n-1)*ICC] (2)

With EF = efficiency factor.

# TREATMENT OF SUBJECTS

## Investigational product/treatment

The intervention is modeled after the flexible stepped-care intervention by van ‘t Veer-Tazelaar et al. ^23^.

The nurse-led stepped-care program consists of four evidence-based treatment steps (see below) lasting 3 months each. In sum, the flow of participants in the intervention group through the stepped-care program depends on their depressive symptom level, measured using the PHQ-9 every 3 months for 1 year. Participants who still have elevated depressive symptom levels after each step are offered participation in the next step. A score below the cut off point results in a period of watchful waiting until an elevated PHQ-9 score indicates the need for the following step of the intervention. By providing stepped care and offering more intensive treatment only to people still having elevated depressive symptom scores, it is expected that patients receive treatment that is tailored to their needs and that available resources are more efficiently used by providing the more intensive treatments only to people who need this. People who meet the MINI diagnostic criteria for depression at baseline or at 3, 6, 9 or 12 months are referred to their GP by the practice nurse. The following treatment steps are offered to participants:

**Step 1:** Watchful waiting. The first 3 months consist of watchful waiting, because depressive symptoms often disappear spontaneously over time. After inclusion and obtaining informed consent, patients are invited by their practice nurse to have an introductory consultation. During this consultation patients will get acquainted with the practice nurse and receive an information brochure that contains information about mild depression and simple advice on how to cope with mild depressive symptoms. Also, patients will be informed about the Stepped-Care program and rationale. In this step, no therapeutic intervention will take place.

**Step 2:** Guided self-help. During this step, participants are offered a self-help course that is specially designed for patients with a chronic physical illness and depressive complaints ^27^. During a visit, the practice nurse will give the patient al necessary material and explain the self-help course. Participants can work through the course at their convenience. In doing so, they will be supported by their practice nurse, who will contact them every other week by phone to monitor their progress. When it is clear that two weeks after receiving the self-help course no activity is undertaken by the patient, the practice nurse will use motivational interviewing techniques by phone to try to activate the patient. When this does not result in more involvement of the patient at four weeks after receiving the self-help course, the practice nurse invites the patient to the practice to discuss the current depressive symptoms (a PHQ-9 will be administered). When depressive symptoms still exist patients are offered to progress early to step 3, where they will receive Problem Solving Treatment (PST)

**Step 3:** problem-solving treatment. In this step, participants are offered Problem Solving Treatment (PST), which is a brief cognitive behavioural intervention that focuses on practical skill building. It consists of a maximum of 7 sessions during which the stages of problem solving are explained and then applied to problems encountered in daily life. The goal of PST is to help patients regain control of their lives ^28 29^.

**Step 4:** Referral to general practitioner (GP). Participants with continuously elevated PHQ-9 scores will be referred to their general practitioner. The participant receives a summary of the treatment received to discuss with their GP.

Participants in the usual care group will have unrestricted access to usual care. Their healthcare uptake (including their use of prescription medications) will be recorded. The practice nurse can consult the GP oubout their patients when they feel the need to. The patients will be informed about this possibility

## Use of co-intervention (if applicable)

Not applicable

## Escape medication (if applicable)

Not applicable, the proposed study does not include research with a medical product.

# INVESTIGATIONAL MEDICAL PRODUCT

# Not applicable

# METHODS

## Study parameters/endpoints

### Main study parameter/endpoint

Primary clinical outcome is the cumulative incidence of DSM-IV major depressive disorder after 12 months (MINI)^30 31^

### Secondary study parameters/endpoints (if applicable)

Secondary clinical outcomes are depression severity (PHQ-9 ^32^ ^33^) and quality of life (EuroQol 5D^34^)

### Other study parameters (if applicable)

In the economic evaluation, health care utilization and absenteeism and presenteeism will be measured using the most recent update of the TiC-P questionnaire^35^. Medication use will be retrieved from the patient’s pharmacy.

Physical outcomes are also measured and include blood pressure, cholesterol and blood glucose (HbA1C).

In the intervention group the uptake of the stepped-care program will also be examined. Process outcome measures that will be studied are the number of contacts with the practice nurse and with the GP, the number of psychotherapy sessions, antidepressant use, number of contacts with the consulting psychiatrist, number of referrals, and patient satisfaction (CSQ^36 37^). Perceived recovery will be measured on a 6-point Likert scale, with answering possibilities ranging from ‘much worse’ to ‘completely recovered’.

To be able to control for confounding variables we will measure the following parameters: Demographics (age, gender, educational level, living situation), severity of anxiety symptoms (HADS-A^38^), Personal and family history of mood disorders trough a subset of the Diagnostic Interview Schedule (DIS)^39^, Dutch questionnaire chronic illnesses^40^, Locus of control^41^ and Social support^42^. All of these questionnaires (except the HADS-A) were previously used in the West-Friesland study^43^.

## Randomisation, blinding and treatment allocation

Randomisation takes place at the level of participating general practices. Before patients are recruited, participating practices will be randomly allocated to serve as intervention practices, where the stepped-care treatment will be implemented, and control practices, where care as usual will be performed. Patients will be allocated to each of the conditions based on the general practice where they are registered. Blinding of patients, GPs and practice nurses is not possible due to the nature of the intervention.

## Study procedures

Practices

All participating general practices will be randomly allocated to either the intervention group, or the care as usual group before the actual start of the intervention. Practice nurses from intervention practices will be trained in the implementation of the stepped care program.

Patients

In all practices, an initial list of adult patients with type 2 diabetes and/or coronary heart disease is composed, using ICPC codes in the GP’s registration system. This list is given to the general practitioner, who excludes all patients that fulfil our preset exclusion criteria, based on the medical file and experiences with the patient. All remaining eligible patients will receive a letter on behalf of the GP, in which all necessary information (at that point) regarding the study, as well as an invitation to participate is included. When patients consider participation, they fill out a screening form with 9 questions (PHQ-9) that will be provided with the information letter and send it back to an indicated address. Patients that did not respond to this initial letter will be sent a reminder. On this screening form, they will be asked for written permission to a.) be contacted by telephone by a researcher to asses whether the patient fulfils the preset inclusion- and exclusion criteriaand to b.) share relevant medical information that is obtained through the screening form and/ or telephone interview with their GP as well.

After returning the PHQ-9 screening form, all patients with a PHQ-9 score of 6 or more are contacted by a researcher to receive additional oral information and to complete the telephone interview. Based on this interview, all patients without a major depression according to the MINI are eligible for the study. These patients receive additional information by mail to obtain informed consent. After receival of the signed informed consent form, patients are included in the Step-Dep trial.

Persons who score less than 6 on the PHQ-9 receive a letter to thank them for their interest in the study,. Patients who already have depression according to the MINI interview are offered an extra opportunity to discuss their sympyoms with their GP. The GP will be informed.

To assure the safety of the patients, we will also inform the GP when the patient seems to have an elevated risk for suicide according to the PHQ-9 (a score of 2 or higher on question 9). From this point, patients are either enrolled in the stepped care program (as described above, p. 14), or will receive care as usual depending on the general practice where they are registered.

Outcomes^[[2]](#footnote-2)^

Patients are asked to complete web-based questionnaires. If they prefer hard copies, we will provide these. In the intervention group the PHQ-9 will also be administered by the nurse in the general practice for clinical monitoring and to be able to adjust the treatment if necessary.

In both groups, part A of the MINI interview (depressive episode) will be administered on the phone at baseline, 6 and 12 months, by qualified research staff. The social support questionnaire and the Dutch questionnaire chronic illnesses will also be administered at baseline, 6 and 12 months. The following parameters will be measured at baseline,3, 6, 9 and 12 months: PHQ-9, EuroQol-5D, HADS-A, Tic-P, perceived recovery and locus of control. Demographics and the subset of the DIS will only be measured at baseline. The CSQ will only be administered at 12 months.Blood pressure, cholesterol and blood glucose (HbA1C), will be measured at baseline and at 12 months follow-up. These measurements are performed as part of the usual care for this group of patients. Therefore, patients undergo no extra procedures for these measurements.

After 24 and 36 months, patients are asked to participate in a long term follop-up. When they agree, they are asked to complete the web-based questionnaires again after 24 and 36 months. Also, part A of the MINI interview will be administered again by phone after 24 and 36 months.

A process evaluation will be performed in which the barriers and facilitators of the stepped care program, and the experiences with the program will be evaluated. This will be done by organizing focus groups among volunteering patients, general practitioners and primary care nurses.

Also after 12 months the uptake of the stepped care program will be evaluated. To do this, the number of contacts with the practice nurse, the number of Problem Solving Treatment sessions, antidepressant use, and the number of referrals to the GP will be assessed in the intervention group by the practice nurse. In the control group, the Tic-p questionnaire will be used to assess the mental health care utilization rates, patient satisfaction will also be evaluated using focus groups.

Burden and Risk

In this study, no ususal diagnostic procedures or treatment will be postponed in either the intervention group or the control group. Also, no invasive measurements are done specifically for this study. When there is an indication that specialised treatment is necessary, patients will be referred to specialised care according to current validated guidelines and protocols. Treatment will not be restricted in any way. Therefore the risk to participate in this trial is close to negligible.

The time needed for patients to complete the questionnaires at baseline, 3, 6, 9 and 12 months is approximately 45 minutes each time. The MINI interviews will be administered three times and will take a approximately 15 minutes each time. In addition, patients in the intervention group will have appointments with the practice nurse. These appointments will take about half an hour to 45 minutes each. The number of appointments a patient in the intervention group gets varies between 5 and 11 and the exact number depends on the individual needs and preferences of the patient. Also, patients in the intervention group who participate in the self-help course will be called about 5 times by the practice nurse to monitor progress and motivate the patient. Such a monitoring call will take about 5 minutes.

Patients in both groups are also asked to participate in a long term follow up at 24 and 36 months. They will be asked to complete the questionnaires, which will again take about 45 minutes per patient for each of the measurements. Also the Mini interview will be administered at 24 and 36 months, again taking about 15 minutes per patient each time.

## Withdrawal of individual subjects

Subjects can leave the study at any time for any reason if they wish to do so without any consequences. The investigator can decide to withdraw a subject from the study for urgent medical reasons.

### Specific criteria for withdrawal

Patients will be withdrawn from the study when they at any point:

- develop suicidal ideation;
- are diagnosed with bipolar disorder; cognitive impairment or dementia,

psychotic illness or terminal illness

## Replacement of individual subjects after withdrawal

Until the sample size for the study has been reached, candidates can participate. Patients will not be replaced. No specific conditions apply for replacing an individual subject after withdrawal. When a patient withdraws him/herself, he/she can continue treatment outside the study.

## Follow-up of subjects withdrawn from treatment

After withdrawal, patients will be asked to keep filling in the questionnaires, because we aim to perform intention to treat analyses.

## Premature termination of the study

Premature termination of the study is possible under the following circumstances.

A) If no positive decision is obtained with regard to the research of if the judgement of the competent medical research ethics committee that has assessed the research is irrevocably is revoked;

B) If a reasonable case can be made for terminating the research in the interest of the subjects’ health;

C) If it transpires that continuation of the research can not serve any scientific purpose, and this is confirmed by the medical research ethics committee that has issued a positive decision on the research;

D) If one of the parties fails to comply with the obligations arising from the agreement and, provided compliance is not permanently impossible, this compliance has not taken place within thirty days after the defaulting party has received a written request to comply, unless failure to comply is out of reasonable proportion to the premature termination of the research.

# SAFETY REPORTING

## Section 10 WMO event

In accordance to section 10, subsection 1, of the WMO, the investigator will inform the subjects and the reviewing accredited METC if anything occurs, on the basis of which it appears that the disadvantages of participation may be significantly greater than was foreseen in the research proposal. The study will be suspended pending further review by the accredited METC, except insofar as suspension would jeopardise the subjects’ health. The investigator will take care that all subjects are kept informed.

## Adverse and serious adverse events

Adverse events are defined as any undesirable experience occurring to a subject during the study, whether or not considered related to the experimental treatment. All adverse events reported spontaneously by the subject or observed by the investiga­tor or his staff will be recorded.

A serious adverse event is any untoward medical occurrence or effect that at any dose:

- results in death;
- is life threatening (at the time of the event);
- requires hospitalisation or prolongation of existing inpatients’ hospitalisation;
- results in persistent or significant disability or incapacity;
- is a congenital anomaly or birth defect;
- is a new event of the trial likely to affect the safety of the subjects, such as an unexpected outcome of an adverse reaction, lack of efficacy of an IMP used for the treatment of a life threatening disease, major safety finding from a newly completed animal study, etc.

All SAEs will be reported through the web portal *ToetsingOnline* to the accredited METC that approved the protocol, within 15 days after the sponsor has first knowledge of the serious adverse reactions..

SAEs that result in death or are life threatening should be reported expedited. The expedited reporting will occur not later than 7 days after the responsible investigator has first knowledge of the adverse reaction. This is for a preliminary report with another 8 days for completion of the report.

**8.2.1 suspected unexpected serious adverse reactions**

Not applicable

**8.2.2 Annual safety report**

Not applicable

## Follow-up of adverse events

All adverse events will be followed until they have abated, or until a stable situation has been reached. Depending on the event, follow up may require additional tests or medical procedures as indicated, and/or referral to the general physician or a medical specialist.

## Data Safety Monitoring Board (DSMB)

A data safety monitoring board (DSMB) will not be assembled for this study. All adverse events, serious or not, unanticipated or not, will be reported to the appropriate ethics and regulatory agencies in accordance with reporting requirements.

# STATISTICAL ANALYSIS

## Descriptive statistics

All patients will be given a distinctive number; data will be analysed anonymously. Baseline data will be presented comparing the two treatment groups.

## Univariate analysis

The analyses will be on an intention-to-treat basis and will include a head-to-head comparison of the stepped care group and the usual care group as well as mixed model analyses. To test the cumulative incidence of depression over time, logistic

mixed model analysis will be used. The obtained odds ratio describes the reduction in the risk of a MINI/DSM-IV depressive disorder in the intervention group relative to the control group. Linear and logistic mixed models (depending on the outcome) will also be used to test differences symptoms of depression an anxiety and quality of life between both groups over time.

## Multivariate analysis

In case of unequal distributions of variables, multivariate analyses techniques will be used to correct for these between-group differences.

The economic evaluation will also be analysed according to the intention-to-treat principle. Missing cost and effect data will be imputed using multiple imputation according to the MICE algorithm developed by Van Buuren ^45^. Costs typically have a highly skewed distribution. Policy makers want to have information on the difference in mean total costs between the two treatment groups to be able to estimate the total health care budget needed for a specific condition.^46^ Therefore, bias-corrected and accelerated

bootstrapping with 5000 replications will be used to calculate 95% confidence intervals around the mean difference in total costs between the treatment groups. Incremental cost-effectiveness ratios (ICERs) will be calculated by dividing the difference in mean total costs between the treatment groups by the difference in mean effects between the treatment groups. Bootstrapping will be used to estimate the uncertainty surrounding the ICERs which will be graphically presented on cost-effectiveness planes. Cost- effectiveness acceptability curves and net monetary benefits will also be calculated.

Cost-effectiveness acceptability curves show the probability that collaborative care is cost-effective in comparison with usual care for a range of different ceiling ratios thereby showing decision uncertainty ^47^.

**9.4 interim analyses**

Not applicable

# ETHICAL CONSIDERATIONS

## Regulation statement

The study will be conducted according to the principles of het Declaration of Helsinki (*WORLD MEDICAL ASSOCIATION DECLARATION OF HELSINKI – Ethical Principles for Medical Research Involving Human Subjects; adopted by the 18^th^ WMA General Assembly, Helsinki, Finland, June 1964, and amended by the 29^th^ WMA General Assembly, Tokyo, Japan, Oktober 1975, 35^th^ WMA General Assembly, Venice, Italy, October 1983; 41^st^ WMA General Assembly, Hong Kong, September 1989; 48^th^ WMA General Assembly, Somerset West, Republic of South Africa October 1996; 52^nd^ WMA General Assembly, Edinburgh, Scotland October 2000; 53^rd^ WMA General Assembly, Washington, United States, (Note of clarification on Paragraph 29 added), 2002; 55^th^ WMA General Assembly, Tokyo, Japan, (Note of clarification on Paragraph 30 added), 2004 and 59^th^ WMA General Assembly, Seoul, October 2008*) and in accordance with the Medical Research Involving Human Subjects Act (*WMO*).

## Recruitment and consent

Patients are recruited at the level of participating general practices. All Patients meeting the initial criteria receive an invitation to participate in the study and to complete the screener for depressive symptoms (PHQ-9) on behalf of their general practitioner. This is necessary, because the identity and personal information of patients initially remain unknown to the research team. However, the invitation letter will be signed also by the research team and in this letter will be clearly stated that the quality and content of care patients receive will not be affected, should they decide not to participate in the study.

Along with this initial invitation all necessary information is provided (Section E3 of the protocol). Upon returning the PHQ-9, patients who have a positive score (≥6) on the PHQ-9 but do not have a depressive disorder according to the MINI interview will be informed further of the purpose and procedures of the study. Each patient receives a general brochure concerning scientific research involving human subjects (in Dutch: medisch-etisch wetenschappelijk onderzoek met mensen), information about the participator insurance and an information letter (Section E1 of the protocol). After giving informed consent by means of a consent form (Section E2 of the protocol), subjects are assigned either to the intervention group or to the control group, depending on the randomisation of their general practice.

## Objection by minors or incapacitated subjects (if applicable)

Not applicable

## Benefits and risks assessment, group relatedness

The screening procedure that will be used in this study potentially offers better recognition of sub threshold depression among adults with DM2 and/or CHD. Furthermore, participating in the proposed stepped-care program will potentially offer better treatment options to patients with DM2 and/or CHD and sub threshold depression in primary care. This is important, since sub threshold depression is very common in these patient groups and might have multiple adverse health effects.

To assess whether the proposed intervention is beneficial and cost-effective for the study population in primary care, it is necessary to collect data trough the administration of several questionnaires.

No major health risks are associated with participating in the proposed study.

## Compensation for injury

The sponsor has a liability insurance which is in accordance with article7, subsection 6 of the WMO. The sponsor also has an insurance which is in accordance with the legal requirements in the Netherlands (Article 7 WMO and the Measure regarding Compulsory Insurance for Clinical Research in Humans of the 23^rd^ of June 2003).

This insurance provides cover for damage to research subjects through injury or death caused by the study.

1. A maximum of €450.000,-- (i.e. four hundred and fifty thousand Euro) for death or injury for each subject who participates in the research
2. A maximum of €3.500.500,-- (i.e. three million five hundred thousand Euro) for death or injury for all subjects for all subjects who participate in the research
3. A maximum of €5.000.000,-- (i.e. five million Euro) for the total damage incurred by the organisation for all damage disclosed by scientific research for the sponsor as ‘verrichter’ in the meaning of said Act in each year of insurance coverage.

The insurance applies to the damage that becomes apparent during the study or within 4 years after the end of the study.

## Incentives (if applicable)

Not applicable

# ADMINISTRATIVE ASPECTS AND PUBLICATION

## Handling and storage of data and documents

Web based questionnaires will be used to administer questionnaires, and an online data management system will be used for the management of these questionnaires. This software is installed on the network of the VU health sciences department, so that backup and access protection is assured.

The participants will receive an email notification when it is time to complete a questionnaire. The research group will monitor compliance with a patient tracking program. This enables the investigators to send out reminders and check whether all questionnaires are completed. If the patient prefers hard copies, they will be provided.

The data that is obtained during the study will be saved apart from any information about the identity of the participants. The obtained data will be coded by a number for the general practitioner that treats the patient and a individual number, starting with 1 for the first participant. The key to the code is safeguarded by the investigator.

The database in which all data from the questionnaires is stored will be safeguarded with tokens and passwords.

The investigators will only receive the data and the data will be processed without knowledge about the treatment group to which participants are assigned. The randomisation of general practices will be done using a computer based list.

## Amendments

Amendments are changes made to the research after a favourable opinion by the accredited METC has been given. All amendments will be notified to the METC that gave a favourable opinion.

Non-substantial amendments will not be notified to the accredited METC and the competent authority, but will be recorded and filed by the sponsor.

## Annual progress report

The sponsor/investigator will submit a summary of the progress of the trial to the accredited METC once a year. Information will be provided on the date of inclusion of the first subject, numbers of subjects included and numbers of subjects that have completed the trial, serious adverse events/ serious adverse reactions, other problems, and amendments.

## End of study report

The investigator will notify the accredited METC of the end of the study within a period of 8 weeks. The end of the study is defined as the last patient’s last visit.

In case the study is ended prematurely, the investigator will notify the accredited METC, including the reasons for the premature termination.

 Within one year after the end of the study, the investigator/sponsor will submit a final study report with the results of the study, including any publications/abstracts of the study, to the accredited METC.

## Public disclosure and publication policy

The principal investigator is free to publish.

# REFERENCES

1. Smits F, Smits N, Schoevers R, Deeg D, Beekman A, Cuijpers P. An epidemiological approach to depression prevention in old age. *Am J Geriatr Psychiatry* 2008;16(6):444.

2. Cuijpers P, Van Straten A, Smit F, Mihalopoulos C, Beekman A. Preventing the onset of depressive disorders: a meta-analytic review of psychological interventions. *Am J Psychiatry* 2008;165(10):1272-80.

3. Mathers CD, Loncar D. Projections of global mortality and burden of disease from 2002 to 2030. *PLoS med* 2006;3(11):e442.

4. Anderson RJ, Freedland KE, Clouse RE, Lustman PJ. The prevalence of comorbid depression in adults with diabetes. *Diabetes care* 2001;24(6):1069-78.

5. Härter M, Baumeister H, Reuter K, Jacobi F, Höfler M, Bengel J, et al. Increased 12-month prevalence rates of mental disorders in patients with chronic somatic diseases. *Psychother. Psychosom.* 2007;76(6):354-60.

6. Rudisch B, Nemeroff CB. Epidemiology of comorbid coronary artery disease and depression. *Biol. Psychiatry* 2003;54(3):227-40.

7. de Graaf R, ten Have M, van Gool C, van Dorsselaer S. Prevalence of mental disorders and trends from 1996 to 2009. Results from the Netherlands Mental Health Survey and Incidence Study-2. *Soc Psychiatry Psychiatr Epidemiol* 2012;47(2):203-13.

8. Kessler RC, Ustun T. *The WHO World Mental Health Surveys*: Cambridge University Press Cambridge, 2008.

9. Bot M, Pouwer F, Ormel J, Slaets JPJ, De Jonge P. Predictors of incident major depression in diabetic outpatients with subthreshold depression. *Diabet. Med.* 2010;27(11):1295-301.

10. Thombs BD, Bass EB, Ford DE, Stewart KJ, Tsilidis KK, Patel U, et al. Prevalence of depression in survivors of acute myocardial infarction. *J. Gen. Intern. Med.* 2006;21(1):30-38.

11. Gehi A, Haas D, Pipkin S, Whooley MA. Depression and medication adherence in outpatients with coronary heart disease: findings from the Heart and Soul Study. *Arch Int Med* 2005;165(21):2508.

12. Lin EHB, Katon W, Von Korff M, Rutter C, Simon GE, Oliver M, et al. Relationship of depression and diabetes self-care, medication adherence, and preventive care. *Diabetes care* 2004;27(9):2154-60.

13. Ruo B, Rumsfeld JS, Hlatky MA, Liu H, Browner WS, Whooley MA. Depressive symptoms and health-related quality of life. *JAMA* 2003;290(2):215-21.

14. Ali S, Stone M, Skinner TC, Robertson N, Davies M, Khunti K. The association between depression and health-related quality of life in people with type 2 diabetes: a systematic literature review. *Diabetes. Metab. Res. Rev.* 2010;26(2):75-89.

15. Lin EHB, Heckbert SR, Rutter CM, Katon WJ, Ciechanowski P, Ludman EJ, et al. Depression and increased mortality in diabetes: unexpected causes of death. *Ann Fam Med* 2009;7(5):414-21.

16. Katon W, Lin EHB, Von Korff M, Ciechanowski P, Ludman E, Young B, et al. Integrating depression and chronic disease care among patients with diabetes and/or coronary heart disease: the design of the TEAMcare study. *Contemp clin trials* 2010;31(4):312-22.

17. Simon GE, Katon WJ, Lin EHB, Ludman E, VonKorff M, Ciechanowski P, et al. Diabetes complications and depression as predictors of health service costs. *Gen Hosp Psychiatry* 2005;27(5):344-51.

18. Rutledge T, Vaccarino V, Johnson BD, Bittner V, Olson MB, Linke SE, et al. Depression and cardiovascular health care costs among women with suspected myocardial ischemia: prospective results from the WISE (Women's Ischemia Syndrome Evaluation) Study. *J. Am. Coll. Cardiol.* 2009;53(2):176-83.

19. Bosmans JE, Adriaanse MC. Outpatient costs in pharmaceutically treated diabetes patients with and without a diagnosis of depression in a Dutch primary care setting. *BMC Health Serv Res* 2012;12(1):46.

20. Smit F, Willemse G, Koopmanschap M, Onrust S, Cuijpers P, Beekman A. Cost-effectiveness of preventing depression in primary care patients. *Br J psychiatry* 2006;188(4):330-36.

21. Cuijpers P, Beekman ATF, Reynolds CF. Preventing Depression. *JAMA* 2012;307(10):1033-34.

22. Muñoz RF, Cuijpers P, Smit F, Barrera AZ, Leykin Y. Prevention of major depression. *Annu Rev Clin Psychol* 2010;6:181-212.

23. van't Veer-Tazelaar PJ, van Marwijk HWJ, van Oppen P, van Hout HPJ, van der Horst HE, Cuijpers P, et al. Stepped-care prevention of anxiety and depression in late life: a randomized controlled trial. *Arch Gen Psychiatry* 2009;66(3):297.

24. Katon WJ, Von Korff M, Lin EHB, Simon G, Ludman E, Russo J, et al. The Pathways Study: a randomized trial of collaborative care in patients with diabetes and depression. *Arch Gen Psychiatry* 2004;61(10):1042.

25. Katon WJ, Lin EHB, Von Korff M, Ciechanowski P, Ludman EJ, Young B, et al. Collaborative care for patients with depression and chronic illnesses. *N Engl J Med* 2010;363(27):2611-20.

26. Arroll B, Goodyear-Smith F, Crengle S, Gunn J, Kerse N, Fishman T, et al. Validation of PHQ-2 and PHQ-9 to screen for major depression in the primary care population. *The Annals of Family Medicine* 2010;8(4):348-53.

27. Voordouw I, van Osch B, Terweij M. De cursus Leven met een chronische ziekte, 2005.

28. Bosmans JE, Brook OH, van Hout HPJ, de Bruijne MC, Nieuwenhuyse H, Bouter LM, et al. Cost effectiveness of a pharmacy-based coaching programme to improve adherence to antidepressants. *Pharmacoeconomics* 2007;25(1):25-37.

29. Mynors-Wallis L, Davies I, Gray A, Barbour F, Gath D. A randomised controlled trial and cost analysis of problem-solving treatment for emotional disorders given by community nurses in primary care. *Br J psychiatry* 1997;170(2):113-19.

30. Sheehan DV, Lecrubier Y, Sheehan KH, Amorim P, Janavs J, Weiller E, et al. The Mini-International Neuropsychiatric Interview (MINI): the development and validation of a structured diagnostic psychiatric interview for DSM-IV and ICD-10. *J Clin psychiatry* 1998;59:22-33.

31. Van Vliet I, De Beurs E. The MINI-International Neuropsychiatric Interview. A brief structured diagnostic psychiatric interview for DSM-IV en ICD-10 psychiatric disorders]. *Tijdschrift voor psychiatrie* 2007;49(6):393.

32. Kroenke K, Spitzer RL. The PHQ-9: a new depression diagnostic and severity measure. *Psychiatr Ann* 2002;32(9):1-7.

33. Wittkampf K, van Ravesteijn H, Baas K, van de Hoogen H, Schene A, Bindels P, et al. The accuracy of Patient Health Questionnaire-9 in detecting depression and measuring depression severity in high-risk groups in primary care. *Gen Hosp Psychiatry* 2009;31(5):451-59.

34. Brooks R. EuroQol: the current state of play. *Health policy* 1996;37(1):53-72.

35. Roijen L, Straten A, Tiemens B, Donker M. Handleiding Trimbos/iMTA questionnaire for Costs associated with Psychiatric illness (TiC-P): Institute of Medical Technology Assessment (iMTA), 2002.

36. De Brey H. A cross-national validation of the client satisfaction questionnaire: the Dutch experience. *Eval. Program Plann.* 1983;6(3):395-400.

37. De Wilde EF, Hendriks VM. The Client Satisfaction Questionnaire: psychometric properties in a Dutch addict population. *Eur. Addict. Res.* 2005;11(4):157-62.

38. Donker T, Van Straten A, Marks I, Cuijpers P. A brief Web-based screening questionnaire for common mental disorders: development and validation. *Journal of medical Internet research* 2009;11(3).

39. Robins LN, Helzer JE, Croughan J, Ratcliff KS. National Institute of Mental Health diagnostic interview schedule: its history, characteristics, and validity. *Arch General Psychiatry* 1981;38(4):381.

40. Kriegsman DMW, Penninx BWJH, Van Eijk JTM, Boeke AJP, Deeg DJH. Self-reports and general practitioner information on the presence of chronic diseases in community dwelling elderly* 1:: A study on the accuracy of patients' self-reports and on determinants of inaccuracy. *J. Clin. Epidemiol.* 1996;49(12):1407-17.

41. Pearlin LI, Schooler C. The Structure of Coping. *J. Health Soc. Behav.* 1978;19(1):2-21.

42. Penninx BWJH, Van Tilburg T, Kriegsman DMW, Deeg DJH, Boeke AJP, van Eijk JTM. Effects of social support and personal coping resources on mortality in older age: the Longitudinal Aging Study Amsterdam. *Am. J. Epidemiol.* 1997;146(6):510-19.

43. Bijl D, Van Marwijk H, Beekman A, De Haan M, Van Tilburg W. A randomized controlled trial to improve the recognition, diagnosis and treatment of major depression in elderly people in general practice: design, first results and feasibility of the West Friesland Study. *Int J Psychiatry Clin Pract* 2003;8(4):135-40.

44. Kroenke K, Spitzer RL, Williams JBW. The Patient Health Questionnaire-2: validity of a two-item depression screener. *Medical care* 2003;41(11):1284.

45. Van Buuren S, Groothuis-Oudshoorn K. MICE: Multivariate imputation by chained equations in R. *J Stat Softw* 2010:1-68.

46. Thompson SG, Barber JA. How should cost data in pragmatic randomised trials be analysed? *BMJ* 2000;320(7243):1197-200.

47. Fenwick E, O'Brien BJ, Briggs A. Cost-effectiveness acceptability curves–facts, fallacies and frequently asked questions. *Health Econ* 2004;13(5):405-15.

# Appendix

Table 1:

Table of measurements

|  | **Meetmoment** | | |  | |  |  | |  |  | |  |  | |  |  | | |  |
| --- | --- | --- | --- | --- | --- | --- | --- | --- | --- | --- | --- | --- | --- | --- | --- | --- | --- | --- | --- |
|  |  | |  |  | |  |  | |  |  | |  |  | |  |  | | |  |
|  | ***screening (-1)*** | | | ***baseline(0)*** | | | ***follow up 3mnd (1)*** | | | ***follow up 6 mnd (2)*** | | | ***follow up 9 mnd(3)*** | | | ***follow up 12mnd (4)*** | | | |
| **Instrument** | **controle** | **interventie** | | **controle** | **interventie** | | **controle** | **interventie** | | **controle** | **interventie** | | **controle** | **interventie** | | **controle** | | **interventie** | |
|  |  |  | |  |  | |  |  | |  |  | |  |  | |  |  | | |
| PHQ-9 per brief | **x** | **x** | |  |  | |  |  | |  |  | |  |  | |  |  | | |
| PHQ-9 per e-questionnaire |  |  | | **x** | **x** | | **x** | **x** | | **x** | **x** | | **x** | **x** | | **x** | **x** | | |
| PHQ-9 door zorgmanager |  |  | |  | **x** | |  | **x** | |  | **x** | |  | **x** | |  | **x** | | |
| MINI psychiatrisch interview (telefonisch door onderzoeker) |  |  | | **x** | **x** | |  |  | | **x** | **x** | |  |  | | **x** | **x** | | |
| Tic-P per e-questionnaire |  |  | | **x** | **x** | | **x** | **x** | | **x** | **x** | | **x** | **x** | | **x** | **x** | | |
| euroquol per e-questionnaire |  |  | | **x** | **x** | | **x** | **x** | | **x** | **x** | | **x** | **x** | | **x** | **x** | | |
| HADS per e-questionnaire |  |  | | **x** | **x** | | **x** | **x** | | **x** | **x** | | **x** | **x** | | **x** | **x** | | |
| DIS per e-questionnaire |  |  | | **x** | **x** | |  |  | |  |  | |  |  | |  |  | | |
| Vragenlijst sociale steun |  |  | | **x** | **x** | |  |  | | **x** | **x** | |  |  | | **x** | **x** | | |
| Vragenlijst chronische ziekten |  |  | | **x** | **x** | |  |  | | **x** | **x** | |  |  | | **x** | **x** | | |
| Locus of control vragenlijst |  |  | | **x** | **x** | | **x** | **x** | | **x** | **x** | | **x** | **x** | | **x** | **x** | | |
| CSQ |  |  | |  |  | |  |  | |  |  | |  |  | | **x** | **x** | | |
|  |  |  | |  |  | |  |  | |  |  | |  |  | |  |  | | |

1. This calculation is adjusted after receiving comments from the EMGO+ Science Committee and ZonMw [↑](#footnote-ref-1)
2. Table of measurements can be found in the appendix [↑](#footnote-ref-2)
